# Bile Acids Do Not Contribute to the Altered Calcium Homeostasis of Platelets from Rats with Biliary Cirrhosis

**DOI:** 10.3389/fphys.2017.00384

**Published:** 2017-06-07

**Authors:** Paola Romecín, Esther G. Navarro, M. Clara Ortiz, David Iyú, Joaquín García-Estañ, Noemí M. Atucha

**Affiliations:** Department Fisiología, Faculty Medicina, Instituto Murciano de Investigación Biosanitaria, Universidad de MurciaMurcia, Spain

**Keywords:** calcium signaling, bile-duct ligation, capacitative calcium entry, cholestasis, liver cirrhosis, fura-2, thapsigargin, thrombin

## Abstract

Previously, we have found that intracellular calcium homeostasis is altered in platelets from an experimental model of liver cirrhosis, the bile-duct ligated (BDL) rat; these alterations are compatible with the existence of a hypercoagulable state and related to an enhanced intracellular calcium release evoked by thrombin and an increased amount of calcium stored in the intracellular organelles. In the present study we have investigated the role of bile acids in those alterations of the BDL cirrhotic model. Cholic acid (CA) or deoxycholic acid (DCA) did not change P-selectin expression or platelet aggregation in any group but elevated baseline platelet calcium levels. Incubation with both bile acids reduced calcium release after stimulation with thrombin in the absence of extracellular calcium. Pretreatment with CA but not with DCA reduced significantly thrombin-induced calcium entry in all three experimental groups. The capacitative calcium entry was also significantly lower in platelets pretreated with both bile acids. The simultaneous addition of thapsigargin and ionomycin to estimate the total amount of calcium in platelet internal stores was decreased by pretreatment with both CA and DCA, although these changes were significantly different in the control rats only with CA and in the BDL platelets with DCA. These results indicate that CA and DCA reduce calcium movements in platelets of control and BDL animals, thus suggesting that bile acids do not participate in the alterations observed in the BDL cirrotic model.

## Introduction

Hypercoagulability in patients with primary biliary cirrhosis has been attributed to elevated fibrinogen and hyperactivity of platelets, which is not observed in non-cholestatic liver diseases, such as chronic hepatitis C, and alcoholic cirrhosis. These changes are believed to be the result of a marked systemic inflammatory activity (Ben-Ari et al., 1997; Papadopoulos et al., 2007). The underlying mechanisms for altered platelet function in cholestasis are unclear. Several mechanisms have been suggested (Bowen et al., 1988; Pihusch et al., 2002; Atucha et al., 2007; Witters et al., 2010; Tripodi et al., 2011), including an intrinsic change in platelet function, e.g., increased aggregation resulting from altered intracellular calcium (Ca^2+^) homeostasis or decreased aggregation due to a storage pool deficiency, the effect of a plasmatic factor such as bile acids or bilirubin or because of the effect of release of ADP-degrading enzymes in the circulation. Regarding platelet activation, the necessary increase in cytoplasmic Ca^2+^ levels, both by release from internal stores and entry of extracellular Ca^2+^, has been reported to be defective both in experimental models of liver cirrhosis and patients (Bandi et al., 1997; Atucha et al., 2007; Annie-Jeyachristy et al., 2008). In previous studies from our laboratory, a greater amount of stored Ca^2+^ together with an increased activity of SERCA, were observed in platelets obtained from a rat model of biliary cirrhosis (Atucha et al., 2007).

The role of bile acids in mediating these alterations in Ca^2+^ signaling and platelet function is not known. Deoxycholic acid (DCA) has been observed to cause profound Ca^2+^ release from intracellular stores and entry from the extracellular medium of intact or permeabilized fibroblast cells (Lau et al., 2005). In diets containing cholesterol and cholic acid (CA), increased thrombin and adenosine diphosphate (ADP)-induced platelet aggregation and an acceleration of the platelet-rich plasma clotting time were found (McGregor et al., 1980). Other authors, however, observed that sodium salts of some bile acids, such as cholic acid, showed a clear inhibitory effect on platelet aggregation by ADP, while the sodium salt of chenodeoxycholic acid (CDA) was an inducer of platelet aggregation (Baele et al., 1980). In other experiments, deoxycholic acid inhibited platelet aggregation and ADP secretion in platelets activated with either collagen or thrombin (Tan et al., 2012).

Thus, in the present study we aimed at analyzing the roles of bile acids such as cholic acid and deoxycolic acid on the altered platelet function observed in a model of cholestasis, the bile duct-ligated rat.

## Materials and methods

### Animals

Male Sprague–Dawley rats born and raised in the Animal House of the Universidad de Murcia were used in the present study. The rats were housed in a temperature controlled environment, with 12:12-h light-dark cycle in the Animal Care Facility of the University of Murcia (REGAES300305440012). The animals were kept and treated according to the guidelines established by the European Union for the protection of animals used in experiments (86/609/EEC). All procedures were approved by the Animal Care and Use Committee of the University of Murcia.

### Experimental groups

Animals weighing ~250 g were subjected to bile-duct ligation (BDL) and excision or sham operation (controls), as described previously (Ortíz et al., 1996; Nadal et al., 2002; Atucha et al., 2003, 2007). Normal rat chow and tap water were offered *ad libitum*. Experiments were performed 21 days later (BDL non-ascitic group) and at least 28 days after surgery, when ascites was present (BDL ascitic group).

### Isolation of platelets

Isolation of platelets were performed as described previously (Iyú et al., 2004; Atucha et al., 2007). Briefly, platelet-rich plasma (PRP) and platelet-poor plasma (PPP) was obtained by differential centrifugation and the platelet count was adjusted to 3. 10^5^ cels/μl.

#### Flow cytometry

##### Determination of platelet activation in PRP through the expression of P-selectin

The expression of P-selectin was performed by flow cytometry in platelets obtained as described above. Briefly, platelets resuspended in PBS were incubated for 30 min with 5 μl of CD61-FITC (for detection of platelet population) and 5 μl CD62P-PE (for detection of P-selectin). The acquisition was set at 50,000 events in the FAC-sort cytometer (Becton-Dickinson). The software used was CELLQuestTM. These experiments were performed in platelets obtained from six animals per group.

### Fura-2 loading and determination of [Ca^2+^]_*i*_

These methods were carried out as described previously (Grynkiewicz et al., 1985; Atucha et al., 2003, 2007; Iyú et al., 2004) in platelets obtained from six rats per group. After obtaining platelet-rich plasma, platelets were washed and incubated with 2.5 μmol/l fura-2/AM (Molecular Probes) for 45 min at room temperature. Then, after washing out fura-2, platelets were stored at room temperature in the dark until Ca^2+^ measurements were performed. Platelets were placed in fluorescence-free cuvettes (Sigma) in the optical field of a fluorescence spectrometer (Aminco Bowman 2; Microbeam), and excited alternatively with light at 340 and 380 nm and the light emitted at 510 nm was collected. Changes in cytosolic free Ca^2+^ concentration ([Ca^2+^]_i_) were obtained by using the fura-2 340/380 fluorescence ratio and calibrated as described previously (Atucha et al., 2007). Only one concentration of each drug was tested on every platelet suspension. The calibration procedure was done in every experiment to take into account differences in the number of platelets between animals. After obtaining baseline values for 30 s, the appropriate drug concentration was added and the fluorescence recorded. Three protocols were performed:

1. The response to thrombin (0.3 U/ml) was studied in the absence (no Ca^2+^ added plus 0.5 mmol/l EGTA) and presence (1 mmol/l CaCl_2_) of extracellular Ca^2+^.
2. Capacitative Ca^2+^ entry (CCE) was determined by incubating platelets in a Ca^2+^-free medium with thapsigargin (1 μmol/l). After 180 s, Ca^2+^ (1 mmol/l) was added and changes in [Ca^2+^]_*i*_ were monitored for another 180 s.
3. Ca^2+^ accumulation into intracellular stores was estimated by suspending platelets in a Ca^2+^-free medium (+100 μmol/l EGTA) and challenging with ionomycin (5 μmol/l) and thapsigargin (1 μmol/l).

In these experiments, to analyze the effect of bile acids, platelets were pre-incubated 10 min at room temperature with CA or DCA at a final concentration of 100 μmol/l). Then, these three protocols were performed again.

### Drugs

All the products used were from Sigma, except where indicated. Fura-2 AM (fura 2 acetoxymethyl ester, Molecular Probes) and Thapsigargin (Invitrogen) were dissolved in DMSO. Appropriate dilutions were prepared freshly every day in measurement buffer (Atucha et al., 2007).

### Statistical analysis

Data are expressed as the mean ± S.E.M. In order to compare the Ca^2+^ responses within the same group, the area under the curve (AUC) of the individual Ca^2+^ responses were calculated by summation of all experimental values (180 s) corrected by substraction of the averaged baseline (estimated during 30 s). The resulting values as well as baseline values were compared between groups by one-way analysis of variance and a Student-Newman-Keuls as *post-hoc* test. A probability level of *p* < 0.05 was considered to be a significant difference.

## Results

The BDL rats used in the present study had the typical features of this model, jaundice, enlarged liver and spleen, and mesenteric edema. Ascites was present in variable amounts only in the animals included in this group. Sham-operated animals did not have any of these alterations.

Baseline P-selectin expression was significantly greater in the BDL group as compared to the controls and the BDL-ascitic rats, and CA or DCA did not affect these values in any group (Table 1). Platelet aggregation, which we found significantly elevated in the BDL non-ascitic rats in a previous paper (Romecín et al., 2017) was not modified by pretreatment with those bile acids (data not shown). Basal platelets Ca^2+^ levels was significantly elevated by incubation with both CA and DCA (Table 2) in platelets of the control animals groups, while in platelets of the BDL rats only DCA elevated basal Ca^2+^. In platelets of BDL rats with ascites, there was no significant effect of bile acids (BA).

**Table 1:** P-selectin expression (arbitrary units) in the experimental groups.

	**Ctrl**	**BDL**	**BDL Asc**
Basal	29.8 ± 1.6	65.8 ± 0.6^*^	26.5 ± 1.3
CA 100 μM	25.4 ± 2.1	62.5 ± 0.8^*^	26.6 ± 1.4
DCA 100 μM	22.7 ± 4.0	65.4 ± 1.1^*^	28.2 ± 1.5

* *p < 0.05 vs. control group*.

**Table 2:** Basal calcium levels (nM) in the experimental groups.

	**Ctrl**	**BDL**	**BDL Asc**
Basal	37.6 ± 6.0	36.4 ± 3.2	39.9 ± 7.0
CA 100 μM	47.2 ± 4.3^+^	46.7 ± 6.1	50.2 ± 9.3
DCA 100 μM	72.5 ± 12.1^+^	65.8 ± 13.4^+^	43.6 ± 7.4

+ *p < 0.05 vs. basal in the same group*.

**Figure 1:**
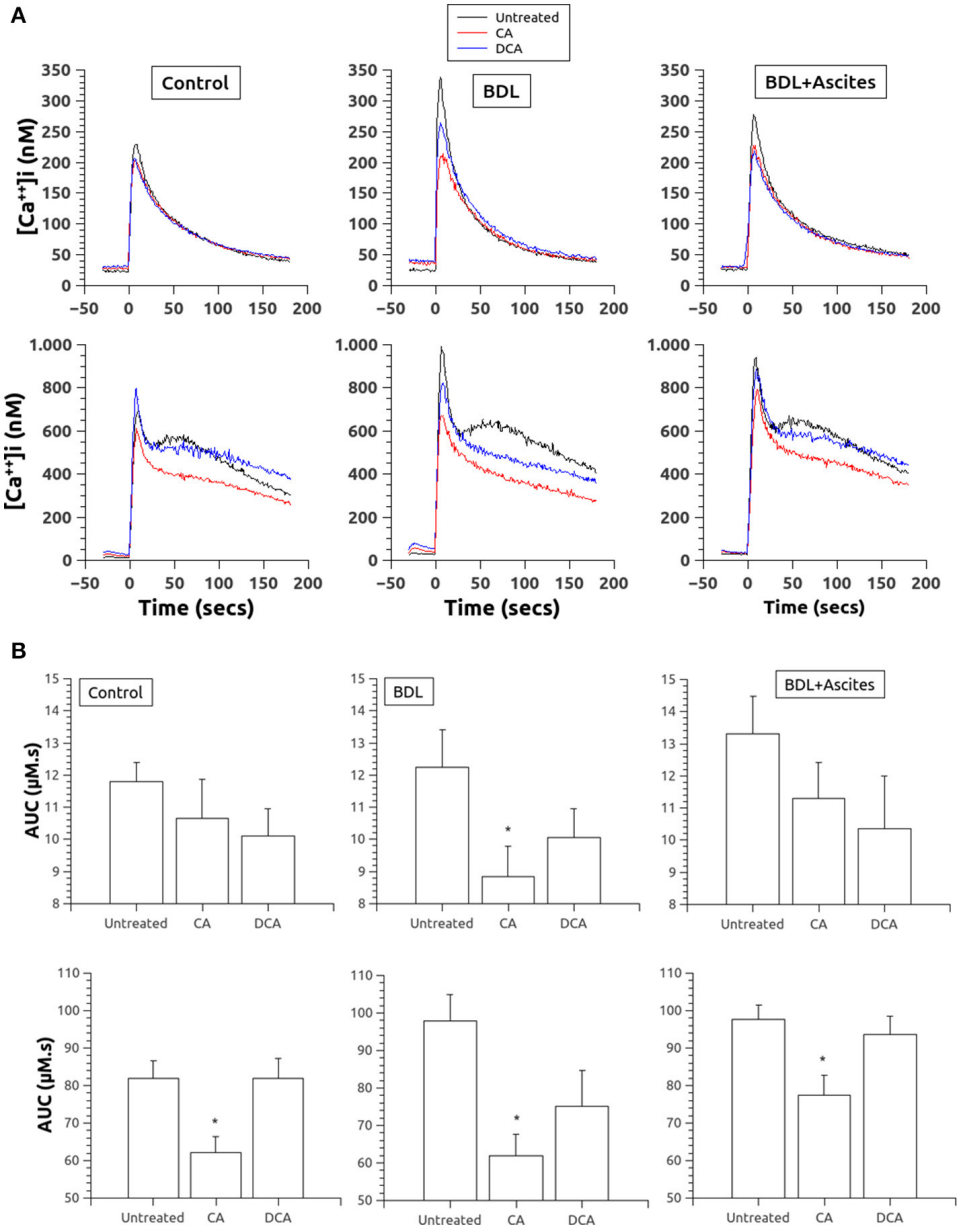
**(A)** Changes in [Ca^2+^]_i_ in platelets of control (left column), BDL (middle column) and BDL with ascites rats (right column) in response to thrombin (0.3 U/ml, added at time 0). Top, in the absence of extracellular Ca^2+^. Bottom, in the presence of extracellular Ca^2+^. Data were obtained in untreated platelets and after incubation with CA or DCA (100 μM each). **(B)**. Area under the curve of the calcium changes after thrombin administration (0.3 U/ml, added at time 0) in the absence (top panels) and in the presence (bottom panels) of extracellular Ca^2+^. Data were obtained in untreated platelets and after incubation with CA or DCA (100 μM each). Results are means ± S.E.M. ^*^p < 0.05 vs. Untreated.

In relation with thrombin responses, incubation with both BA reduced the Ca^2+^ release after stimulation with thrombin (0.3 U/ml) in the absence of extracellular Ca^2+^ (Figure 1A, top panels), but only CA in the BDL group showed a significant difference as compared to its basal (Figure 1B, top panels). In the presence of extracellular Ca^2+^ (Figure 1A, bottom panels), thrombin responses were much greater in all three groups and pretreatment with CA but not with DCA, reduced significantly those Ca^2+^ responses in all three experimental groups (Figure 1B, bottom panels).

**Figure 2:**
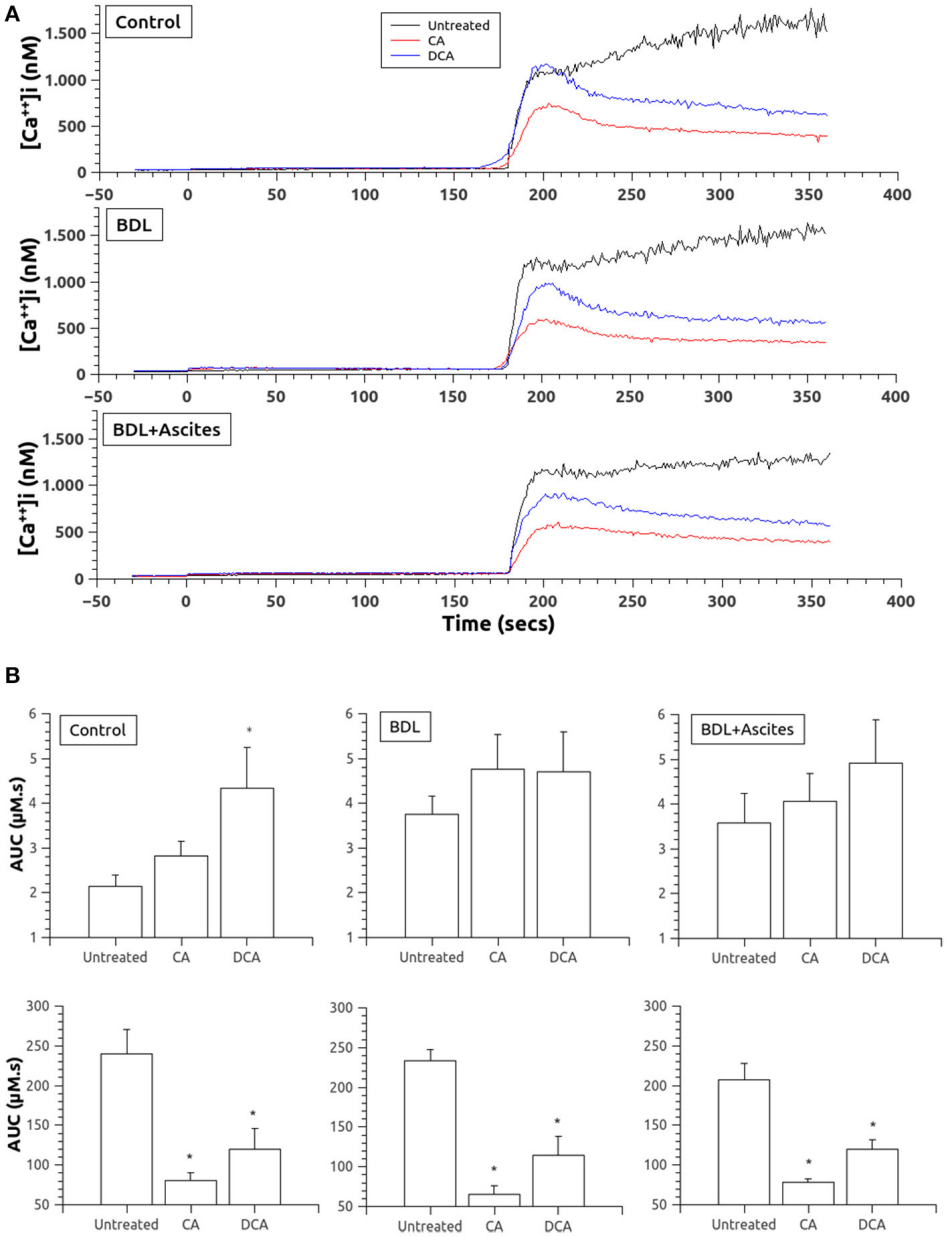
**(A)** Changes in [Ca^2+^]_i_ after thapsigargin administration in the absence of extracellular Ca^2+^ (time 0 seconds) and after addition of calcium to allow for capacitative Ca^2+^ entry (time 180 s). Data were obtained in untreated platelets and after incubation with CA or DCA (100 μM). **(B)** Area under the curve of the calcium changes after thapsigargin administration in the absence of extracellular Ca^2+^ (top panels) and after addition of calcium to allow for capacitative Ca^2+^ entry (bottom panels). Data were obtained in untreated platelets and after incubation with CA or DCA (100 μM). Results are means ± S.E.M. ^*^p < 0.05 vs. Untreated.

The capacitative Ca^2+^ response is shown in Figure 2. Pretreatment with thapsigargin to deplete Ca^2+^ from the intracellular stores slightly elevated Ca^2+^ levels, being significantly greater in the platelets of the BDL rats. However, regarding the effect of BA, only DCA significantly elevated the thapsigargin effect in platelets of the control rats (Figure 2B, top panels). But, the addition of Ca^2+^ in those platelets pretreated with thapsigargin to evoke the capacitative Ca^2+^ entry induced a significantly lower entry of Ca^2+^ in platelets pretreated with both CA and DCA (Figures 2A,B, bottom panels) in all three experimental groups.

**Figure 3:**
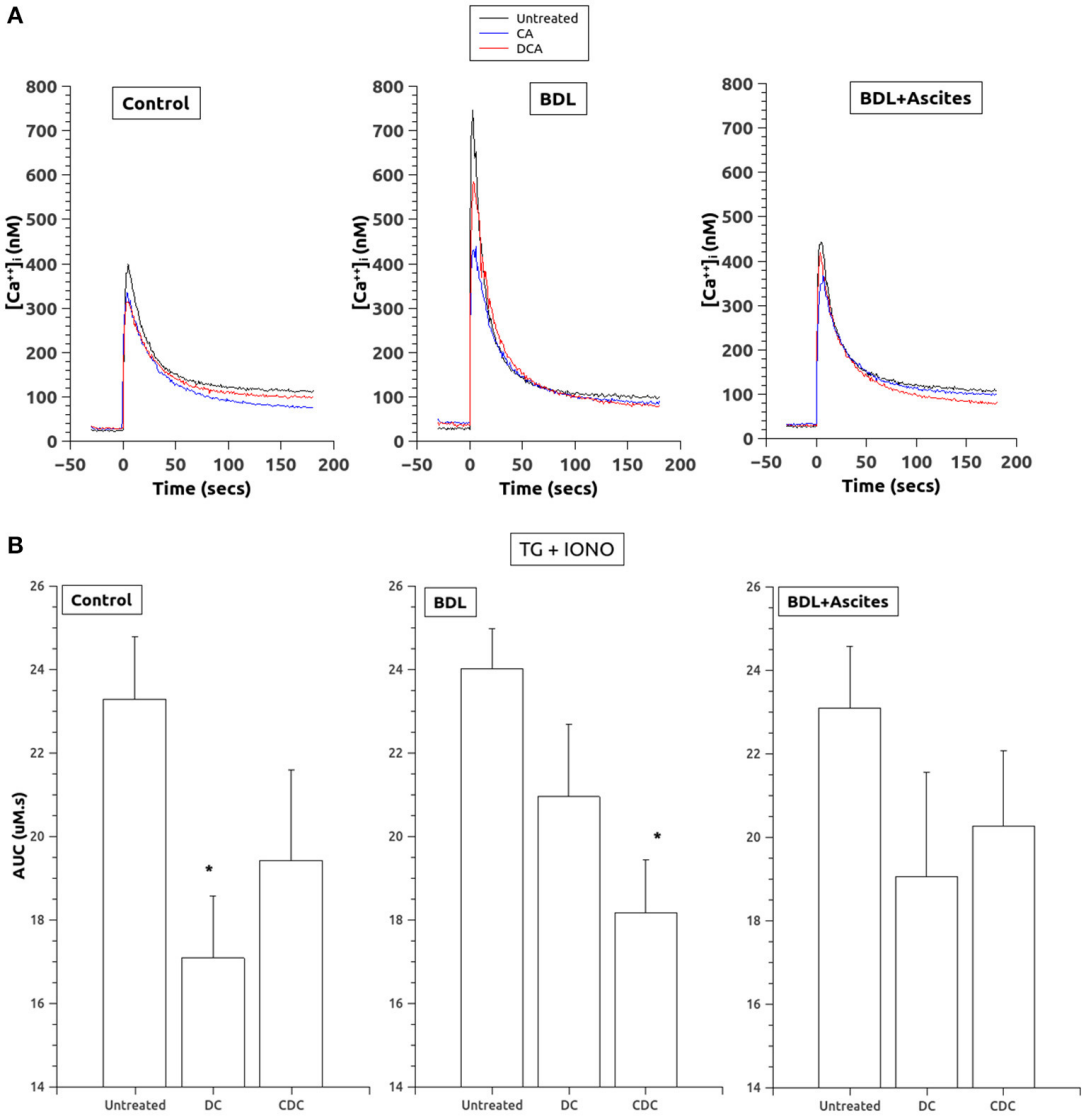
**(A)** Changes in [Ca^2+^]_*i*_ after thapsigargin and ionomycin administration (added at time 0) to allow for estimation of total calcium in intracellular stores. Data were obtained in untreated platelets and after incubation with CA or DCA (100 μM). **(B)** Area under the curve of the calcium changes after thapsigargin and ionomycin to estimate total calcium in intracellular stores. Data were obtained in untreated platelets and after incubation with CA or DCA (100 μM). Data are means ± S.E.M. ^*^p < 0.05 vs. Untreated.

To estimate the amount of Ca^2+^ accumulated into intracellular stores, we used the simultaneous addition of thapsigargin and ionomycin. As shown in Figures 3A,B, total Ca^2+^ stored was significantly decreased by pretreatment with both CA and DCA, although these changes were significantly different in the control rats only with CA and in the BDL platelets with DCA.

## Discussion

In previous studies (Atucha et al., 2007; Romecín et al., 2017), we showed in a rat model of liver cirrhosis by bile duct ligation, several platelet alterations compatible with the existence of a hyperaggregatory state. These alterations are related to a defective platelet Ca^2+^ handling, specifically to an enhanced intracellular Ca^2+^ release evoked by thrombin and an increased amount of Ca^2+^ stored in the intracellular organelles and are present before the appearance of ascites. In the present study we confirm these alterations and extend them to show their lack of relationship to bile acids. Our results indicate that, if any, bile acids, would tend to reduce Ca^2+^ movements across platelet membranes which would reduce this hyperaggregatory state characterisitic of the non-ascitic phase of the disease. A similar result would also occur in the ascitic phase, characterized in this case by a lower platelet aggregatory function.

This hyperaggregatory state is related firstly to a higher expression of P-selectin in both groups of cirrhotic rats, indicative of an activated state, which is typical of several types of human cirrhosis (Panasiuk et al., 2001; Vardareli et al., 2007; Xianghong et al., 2013). These data are similar to those found by Watanabe et al. (1998), in cirrhotic patients, who explained it by a change in the fatty acid composition of the platelet membrane. Although bile acids are well known detergents that exhibit membrane-damaging properties against a variety of cells, including blood platelets (Shiao et al., 1993), this does not seem the case, since in our data the expression of P-selectin was unchanged by pretreatment with CA and CDA.

Regarding thrombin-induced intracellular Ca^2+^ changes, both bile acids clearly decreased both Ca^2+^ release from internal stores (that observed in the absence of Ca^2+^) and Ca^2+^ entry from the extracellular space, although only CA, but not DCA, showed a significantly lower response in all three groups, control and BDL-ligated rats. This result agrees with previous data (Bowen et al., 1988; Shiao et al., 1993) but disagrees with those of McGregor (McGregor et al., 1980) showing that cholic acid increased thrombin and ADP-induced platelet aggregation and accelerated plasma clotting time. A similar result was obtained in fibroblasts, where DCA directly induced both Ca^2+^ release from internal stores and persistent entry at the plasma membrane (Lau et al., 2005).

Our results are in keeping with more recent data indicating that bile acids also act as regulatory molecules (Hylemon et al., 2009). Bile acids have been discovered to activate specific nuclear receptors such as farnesoid X receptor (FXR) and others, as well as cell signaling pathways in cells of the liver and gastrointestinal tract. A recent study by Moraes et al. (2016) indicates that FXR ligands inhibited the activation of platelets in response to stimulation of collagen or thrombin receptors, resulting in diminished intracellular Ca^2+^ mobilization, secretion, fibrinogen binding, and aggregation. However, bile acids also activate a number of cellular Ca^2+^ related channel receptors (Dopico et al., 2002; Lee et al., 2012; Rainer et al., 2013). Since Ca^2+^ regulation is critical for multiple platelet functions (Sage, 1997; Rosado and Sage, 2002; Dolan and Diamond, 2014), we suggest that bile acids, acting through platelet receptors, induce inhibition of platelets by reducing Ca^2+^ signaling. Clearly, more experiments would be necessary in order to prove this hypothesis.

A similar effect of bile acids was found when we studied the capacitative Ca^2+^ entry, again suggesting an interference with the channels involved in those responses. Interestingly, bile acids did not reduce the intracellular Ca^2+^ elevation induced by thapsigargin, an inhibitor of SERCA Ca^2+^ ATP-ase, that although modest compared to the capacitative entry, was significantly elevated with DCA in the platelets of the control animals.

Finally, total amount of Ca^2+^ in internal stores, as estimated with the use of thapsigargin and ionomycin, was again reduced by the bile acids, suggesting again that bile acids interfere with Ca^2+^ signaling receptors both at internal and cellular membranes. Clearly, further studies will be necessary to analyze the platelets receptors involved in these alterations.

## Conclusions

Patients with liver cirrhosis and experimental models show, in the compensated phase prior to the development of ascites, platelet alterations compatible with a hyperaggregatory state. This is related to enhanced Ca^2+^ release evoked by agonists in activated platelets, secondary to an increased amount of Ca^2+^ stored in the intracellular organelles. These alterations are not related to bile acids, since they reduce the amount of internal Ca^2+^ and the entry from the extracellular space, both necessary to induce platelet aggregation.

## Author contributions

PR was the main performer of most of the experiments, EN is our lab technician and without her nothing can be done, MO and DI played a key role with the flow cytometry technique, JG was the main writer of the manuscript and NA supervised all the experimental protocols and procedure laboratories and directed the research.

### Conflict of interest statement

The authors declare that the research was conducted in the absence of any commercial or financial relationships that could be construed as a potential conflict of interest.

## Abbreviations

[Ca^2+^]_*i*_: cytosolic calcium levels
Ca^2+^: calcium
BDL: bile-duct ligated rat
ADP: adenosine diphosphate
CCE: capacitative calcium entry
SERCA: smooth endoplasmic reticulum calcium ATP-ase.
